# Stimuli-responsive transdermal microneedle patches

**DOI:** 10.1016/j.mattod.2021.03.012

**Published:** 2021-05-20

**Authors:** Pooyan Makvandi, Rezvan Jamaledin, Guojun Chen, Zahra Baghbantaraghdari, Ehsan Nazarzadeh Zare, Concetta Di Natale, Valentina Onesto, Raffaele Vecchione, Jesse Lee, Franklin R. Tay, Paolo Netti, Virgilio Mattoli, Ana Jaklenec, Zhen Gu, Robert Langer

**Affiliations:** 1 Istituto Italiano di Tecnologia, Centre for Materials interfaces, Viale Rinaldo Piaggio 34, 56025 Pontedera, Pisa, Italy; 2 Center for Advanced Biomaterials for Health Care (iit@CRIB), Istituto Italiano di Tecnologia, Naples, 80125, Italy; 3 Department of Chemical, Materials & Industrial Production Engineering, University of Naples Federico II, Naples, 80125, Italy; 4 Department of Bioengineering and California NanoSystems Institute, University of California, Los Angeles, CA, 90095, USA; 5 Department of Biomedical Engineering, and the Rosalind & Morris Goodman Cancer Research Centre, McGill University, Montreal, QC, H3G 0B1, Canada; 6 School of Chemistry, Damghan University, Damghan, 36716-41167, Iran; 7 College of Graduate Studies, Augusta University, Augusta, GA, 30912, USA; 8 David H. Koch Institute for Integrative Cancer Research, Massachusetts Institute of Technology, Cambridge, MA, 02139, USA; 9 Jonsson Comprehensive Cancer Center, University of California, Los Angeles, California, 90095, United States; 10 College of Pharmaceutical Sciences, Zhejiang University, Hangzhou, 310058, PR China

## Abstract

Microneedle (MN) patches consisting of miniature needles have emerged as a promising tool to perforate the *stratum corneum* and translocate biomolecules into the dermis in a minimally invasive manner. Stimuli-responsive MN patches represent emerging drug delivery systems that release cargos on-demand as a response to internal or external triggers. In this review, a variety of stimuli-responsive MN patches for controlled drug release are introduced, covering the mechanisms of action toward different indications. Future opportunities and challenges with respect to clinical translation are also discussed.

## Introduction

Conventional routes of drug administration, such as oral and parenteral routes, often have limitations due to the drug’s physicochemical properties. For instance, oral administrations are associated with the first-pass metabolism, which greatly reduces the bioavailability reduce bioavaibility of delivered therapeutics. Injection is invasive, which generally requires administration by trained personnel and is usually painful and potentially associated with needle phobia in patients, especially children [1,2]. Conversely, microneedles (MNs) are capable of penetrating the outermost layer of the skin (*stratum corneum*) to locally administer bioactive molecules into the epidermis and/or superficial dermis. Microneedles offer an appealing administration approach to patients as this method of administration is painless and may be self-administered [3–8].

**FIGURE 1:**
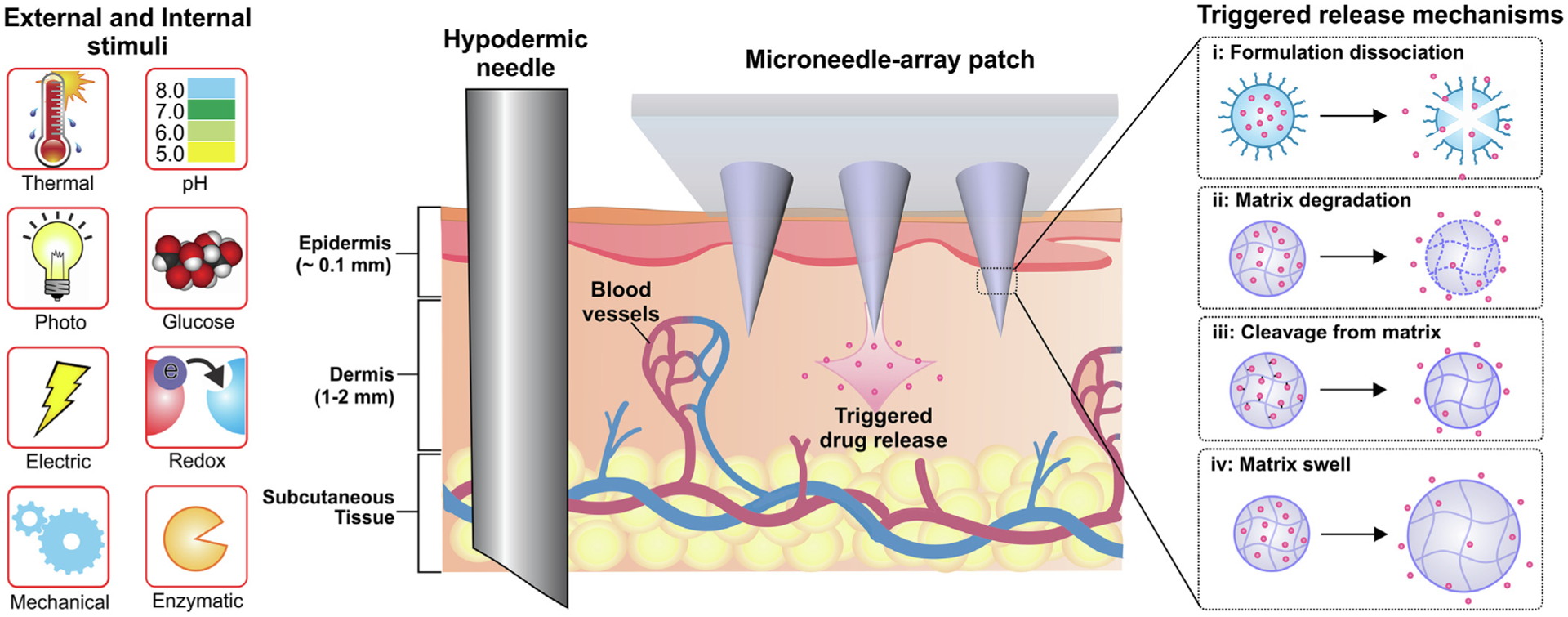
The different types of internal and external stimuli and trigger release mechanisms for stimuli-responsive transdermal MN patch systems.

Recently, stimuli-responsive MNs, typically based on polymeric matrices, have been introduced to facilitate and control the release of payloads [7,9]. Stimuli-responsive materials comprise a wide range of compounds that are capable of responding to changes in their surrounding environment (Fig. 1) [10,11]. The responses of stimuli-responsive materials to the environment may be in the form of formulation dissociation, matrix degradation, cleavage from matrix, and matrix swell [12,13]. These systems release payloads in response to physiological signals as internal stimuli (e.g., pH, redox potential, glucose and enzymes) [14,15] and/or physical signals as external stimuli (e.g., temperature, electric field, light, and mechanical stress) [16–18]. Spatiotemporal control of responsive systems improves therapeutic efficacy and reduces potential side effects associated with off-target drug delivery.

In the present review, the chemistry, structure and functionality of stimuli-responsive MNs are presented. Then responses of these MNs to internal and external stimuli are highlighted along with their current applications and potential future applications in the biomedical field.

## Microneedles triggered by external stimuli

Many efforts have been made in recent years to develop external stimuli-responsive MNs [19,20]. The construction of responsive MNs may require incorporation of domains that can be activated by external stimuli. In the following section, the functionality and structure of responsive compounds employed in externally-triggered stimuli-responsive MNs are introduced along with their biomedical applications. Table 1 summarizes representative responsive MNs to external triggers and their potential in biomedical applications.

### Light-activated MNs

Light has beneficial features such as being non-invasive with high spatial resolution and temporal control. Light-responsive materials are capable of responding to light and are mostly employed for chemotherapy and photothermal therapy. These systems are produced by embedding photosensitive metal/metal oxide nanoparticles (e.g., LaB_6_, Pt, Au, and TiO_2_ nanoparticles) or photochromic agents (e.g., melanin as a photosensitizer) into the polymeric MNs [17,21].

**FIGURE 2:**
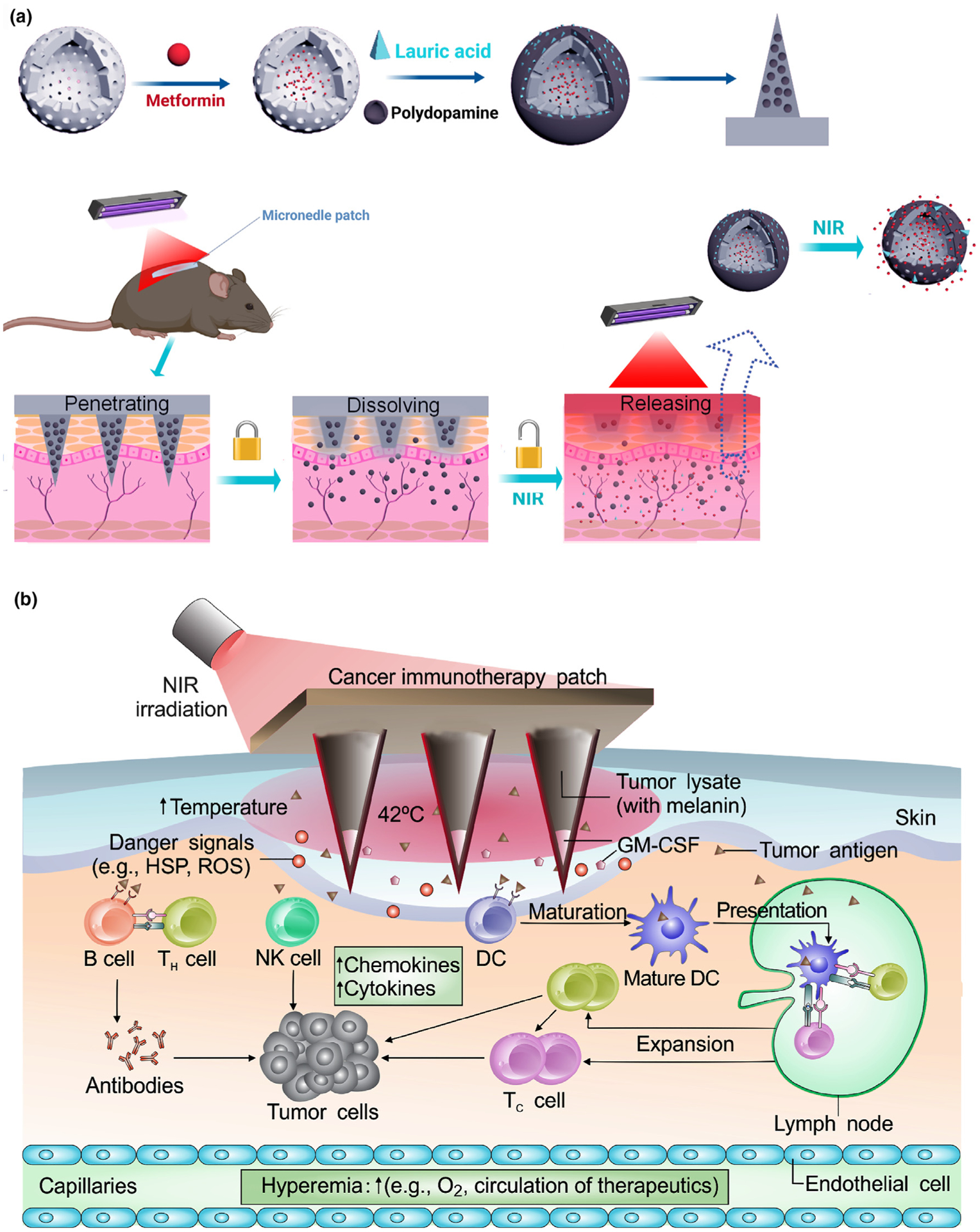
(a) Schematic of the preparation of metformin-loaded hollow mesoporous SiO_2_ (met/HMSN) and decoration of polydopamine (PDA) and lauric acid (LA) on the nanoparticles. Near-infrared (NIR)-responsive release of the loaded metformin on diabetic rats by the transdermal delivery is illustrated. Reproduced with modification from [23]. (b) Illustration of melanin-mediated cancer immunotherapy through a transdermal MN vaccine patch. The presence of melanin, the natural-occurring pigment in the whole tumor lysate, leads to the local release of heat via controllable near-infrared light emission. HSP: heat shock protein; ROS: reactive oxygen species; GM-CSF: granulocyte–macrophage colony-stimulating factor; NK cell: natural killer cell; DC: dendritic cell. Reprinted with permission from [24].

The most commonly applied electromagnetic wave for light-activated MNs is near-infrared (NIR) radiation. Light-activated materials respond through different mechanisms. The first group of light-activated MNs contains polymers with a low melting temperature that can be melted to release their contents. These types of devices are categorized as light-activated dissolvable MNs. These phase-change polymers are generally combined with a photothermal conversion agent (Fig. 2). The temperature for melting polymers may be enhanced by incorporation of metal-based (nano)materials. For instance, LaB_6_ generates heat in response to NIR irradiation and has been used in light-activated MNs [17]. Microneedles based on LaB_6-_polycaprolactone composites demonstrate on-demand release by liquefaction of the polycaprolactone when exposed to an external NIR radiation source [22]. Light-sensitive materials may also potentially be used for treatment of diabetes, in which drug release is controlled by the irradiation source. For example, metformin encapsulated-hollow mesoporous SiO_2_ nanoparticles have been decorated with polydopamine and lauric acid. Polydopamine is employed as the photothermal conversion agent and lauric acid as the phase-change material. Because of polydopamine, the lauric acid coating layer (melting point ≈ 44–46 °C) is melted after exposure to NIR irradiation. The mesopores on SiO_2_ are opened for release of the loaded drug by diffusion. In the absence of NIR laser irradiation, there was only a small amount of metformin released via the drug diffusion route. However, extensive metformin release could be detected after five NIR laser on/off cycles. Such an NIR-triggered MN patch exhibits a low risk of hypoglycemia and no *in vivo* toxicity (Fig. 2A) [23].

Apart from the intrinsically light-responsive substances, chemical coupling of photo-cleavable compounds may be used to synthesize light-activated prodrugs. As these systems utilize inactive prodrugs, covalent bond cleavage regulated by light irradiation is employed to liberate of the encapsulated bioactive drug [25]. For example, conjugation of various drugs (e.g., acetylsalicylic acid, ibuprofen, ketoprofen) to photo-activatable compounds (e.g., dimethylaminopyridine) has been used to endow light responsivity to those drugs [26]. The conjugated prodrug is cleaved released from MNs [27].

The last group of light-activated MNs perform their function by converting light to heat and increasing the surrounding temperatures. For example, a melanin patch has been designed for photothermal therapy (PTT)-mediated enhanced cancer immunotherapy [28]. For example, a patch containing the tumor lysate of melanoma (with melanin) has been made for NIR PTT (Fig. 2B). The patch with tumor-lysates, in combination with NIR-PTT-mediated tumor cell deaths promoted tumorassociated antigen uptake by dendritic cells and resulted in enhanced anti-tumor immune responses. Compared with control mice that were treated with either a MN patch containing only melanin, or a MN loaded only with hyaluronic acid with NIR irradiation, or a vaccine MN patch without NIR irradiation, a vaccine MN patch with NIR irradiation showed significant repression of tumor growth when compared to control groups [24].

### Electric-responsive MNs

Electric-responsive MNs are prepared by incorporation of inherently-conducting polymers such as polyaniline, polyethylene dioxythiophene, and polypyrrole, or conductive nanoarchitectures such as metal nanoparticles and carbon-based nanomaterials [29,30]. An electrically-controlled MN designed for drug release should enable spatiotemporal control of drug secretion. This is critical for alleviating some of the concerns associated with systemic dosing [31].

Inherently-conducting polymers release their contents using electricity as an external stimulus [32]. For instance, polypyrrole has been electrodeposited on MNs to release dexamethasone. This system potentially alleviates swelling in the spinal cord and traumatic brain injury [30]. The manner in which an electric current is delivered to patients to activate the patches is a critical issue to be considered and should be addressed with *in vivo* testing prior to their clinical applications. In a study, MNs transdermal patches based on electro-conductive polypyrrole were designed for electrically-controlled release of dexamethasone for targeting the nervous system. Dexamethasone was electrically secreted from the MNs into wells containing cells by applying electricity (−1 V) for 2 min. This resulted in mitigated free radical production in an *in vitro* model of neuroinflammation [30].

### Magnetic-responsive MNs

Magnetic-responsive MN systems can be fabricated by incorporating nanostructures with magnetic characteristics in the desired polymer matrices [33–37]. Upon injection into the human body, they may be manipulated by an external magnetic field gradient, which allows NPs to be guided towards target regions.

Microneedles with magnetic properties are typically used for detection in bioimaging. For instance, biodegradable magnetic-responsive MN patches have been synthesized using chitosan embedded with magnetic graphene quantum nanodots [36]. Magnetic graphene quantum nanodots (MGQDs) were made by coating the surface of graphene oxide with multiple iron oxide NPs. MGQDs amplifed electrical conductivity and facilitated the biodegradation of chitosan. Microneedles with MGQD nanocomposites have great potential as a drug carrier and dualimaging probe.

### Thermoresponsive MNs

Thermoresponsive MNs are prepared with materials with a low melting temperature (<55 °C), which enable them to undergo solid-to-liquid transition on stimulation by a heat source. These MNs can receive heat from two sources to undergo phase transition. The first heat source is derived from a thermal heater. Polymers such as polycaprolactone with a low melting point (50 °C) undergo phase transition to cause drug release. For example, metformin was embedded in polycaprolactone arrowheads for treatment of type II diabetes. After exposing the MNs to an electroheating sheet (heat source) for a brief period, the polycaprolactone arrowheads underwent phase transition to the liquid state, liberating the encapsulated metformin [25]. The second heat source utilizes photothermal agents that are capable of converting light to localized heat to melt the polymer.

### Mechanical force-responsive MNs

Mechanical forces, including compressive, tensile and shear forces exist ubiquitously within the body and are easily applied externally [38]. Mechanical force-responsive delivery systems, including MN patches, have received increasing attention and offer a convenient route for controlled drug release. The mechanical force-mediated triggers also enable prompt release of therapeutics by patients in a self-administered manner. These MN systems respond to mechanical strains through simple body motions, such as tension in muscles, organs, tendons and bone joints, to promote drug release spatiotemporally. For instance, a stretchable MN patch was fabricated using an elastomer film with microgel depots that contained drug-loaded NPs [18]. Once a tensile strain was applied, drug release from microgel depots was promoted due to the increased surface area of microgel depots and Poisson’s ratio-induced compression on the depots. *In vivo* performance of insulin-loaded MNs was evaluated in type I diabetic mice. When stretched, this stretchable MN patch reduced the blood glucose level to a normal glycemic state within 30 min. Clinically, stretchable MNs containing microdepots may be adapted to the finger joint of a patient to release a therapeutic in response to finger flexion. As a result, daily body movements and intentional movement can provide continuous and pulselike release, respectively. Such a MN device is capable of subcutaneous release of various therapeutics such as anti-inflammatory, anti-infective agent, and analgesics [18].

Another mechanical force-responsive MN system possesses a detachable tip that is separable from the base upon the application of mechanical forces. Because the elasticity of skin causes difficulties for complete insertion of MNs, tips integrated on a substrate can be a promising method to solve this issue. The tip can release drugs based on three mechanisms, including dissolution (e.g., hyaluronic acid tip), melting (e.g., tips made of polymers with low melting points) or degradation (e.g., poly (lactic-co-glycolic acid) tip). Insertion-responsive or tipseparable MNs have been prepared using water-soluble hyaluronic acid (HA) tips and a PCL base for coating vaccine [39]. After penetrating the skin, the HA tips can be separated from the PCL base due to the relatively weak adhesion strength between HA and PCL. The presence of a wall on the PCL base significantly increases separation force and mechanical stability. This prevents tip separation during transport or storage.

Microneedles with other release mechanisms, such as melting, have been developed by utilizing a separable PCL tip built onto a polyvinylpyrrolidone/polyvinyl alcohol base [25]. In the presence of NIR light or electroheating sheet, the PCL tips melt in the skin and release the loaded therapeutics [40].

## Microneedles triggered by internal stimuli

These MNs incorporate polymers that are sensitive to the emergence or variation of a biological signal (e.g., pH, reactive oxygen species (ROS), glucose or enzyme). Based on the pathology involved, researchers can choose different internal triggers. For example, they can take advantage of physiological signals that are typical of tumor niches (e.g., reducing conditions, lower pH and over-expression of enzymes and compounds) to trigger drug release. Moreover, the use of sophisticated devices can be avoided to minimize the cost of the treatment [41]. Table 2 summarizes representative bioresponsive MNs and their potential biomedical applications.

### pH-responsive MNs

pH-responsive MNs are made of polymers that respond to pH variations of the surrounding environment through degradation, swelling or collapsing. These materials typically possess hydrophilic and ionic functional groups in the polymer segments.

Changes in pH in different organs or low pH at the site of chronic wounds/inflammation/cancer have been extensively explored as the stimulus for designing pH-sensitive drug delivery systems [13,42,43]. This attribute has practical implications in cancer therapy because the microenvironment of most tumors has a pH that is lower than non-cancerous tissues [44,45]. Additionally, the mild acidic nature of epidermis can potentially be utilized as a stimulus for self-regulated local drug delivery [46,47]. pH-sensitive polymers are capable of responding to alterations in environmental pH by inducing structural changes in the polymer network, such as degradation, swelling or collapse. These polymers could be utilized as reservoirs in which the drugs are released upon disassembly/deswelling associated with pH changes [48,49].

Because of the acidic environment of the epidermis, pH-responsive carriers may be incorporated in MNs to release a therapeutic specifically to the skin in a controlled manner. For example, a microneedle patch filled with pH-responsive poly(lactic-co-glycolic acid) (PLGA) hollow microspheres was reported for transdermal drug delivery [19]. A model drug (cyanine5) was chosen to be delivered to the skin by polyvinylpyrrolidone (PVP) microneedles. Cyanine5 was encapsulated in PLGA hollow microspheres (HMs) containing sodium bicarbonate (NaHCO_3_). As the MNs were inserted into the skin, the PLGA HMs were liberated while PVP was dissolved in the skin. After a short delay in time, protons in the acidic environment of the skin reacted with NaHCO_3,_ leading to the formation of carbon dioxide. The CO_2_ bubbles generated positive pressure inside the PLGA HMs and pierced the membranes of the microspheres to release the cyanine5.

**FIGURE 3:**
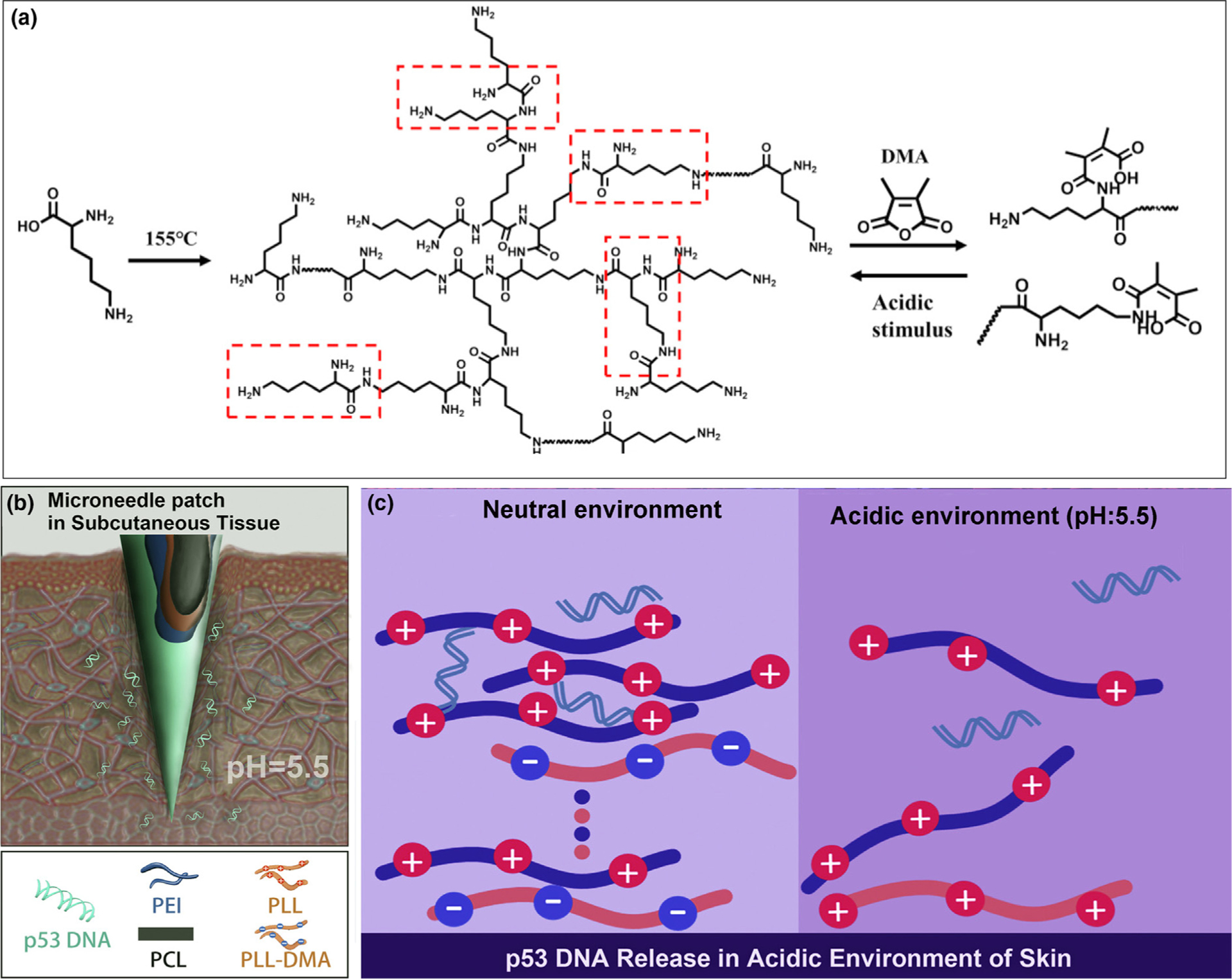
pH-responsive multilayer gene delivery microneedle. (a) Polylysine (PLL) and dimethylmaleic anhydride-modified polylysine (PLL-DMA) synthesis. Hyperbranched PLL, a positively-charged amino-rich polymer, is synthesized from lysin under high temperature. When exposed to DMA, the majority of amino groups form bonds with DMAt, producing PLL-DMA that is rich in –COOH functional groups. In acidic conditions, the amide groups are unstable, and PLL-DMA returns to hyperbranched PLL. Charge alteration here is responsible for pH sensitivity of this system. (b) The polyelectrolyte multilayer coating on the surface of microneedles consists of two parts; a pH-sensitive part composed of PLL-DMA/polyethylenimine (PEI) and a therapeutic agent part consisting of p53DNA/PEI. (c) Close-up of the transition polymer layers on the microneedle surface with gene-containing chains on top. Chemistry of the phase transition process is illustrated in detail. At neutral pH, PLL-DMA expresses a negative charge because of its –COOH groups. At low pH, the polymer transforms into positive-charge hyperbranched PLL. The PLL chain shares the same charge as PEI, which causes collapse of the microneedle and release of the therapeutic cargo within the acidic environment of a tumor. Reprinted with permission from [50].

Because of the acidic nature of cancerous skin, pH-sensitive MNs are employed for targeted delivery of anti-tumor therapeutic agents [48]. A polybasic pH-responsive polymer was deposited as polyelectrolyte multilayers on PCL microneedles to facilitate gene release in the treatment of skin cancer (Fig. 3) [50]. The pH-responsive polymer underwent charge conversion when exposed to the acidic tumor microenvironment. Because of the repulsion of positively-charged groups, the polymeric layers swelled and released the gene cargos. *In vivo* murine experiments showed that experimental mice treated with the pH-responsive MNs had minimal increase in tumor mass, compared to 5- or 8-fold increase in tumor mass in mice treated with non-pH-responsive MNs or with intravenous injection, respectively.

Apart from MNs that respond to low pH values, pH-responsive MN patches may be designed to be responsive to physiological pH values. The principle behind this strategy is based on manufacturing MNs at low pH, so that they respond upon insertion into skin with physiological pH [51]. A pH-responsive MN patch that responds to physiological pH has been used for improving the efficacy of DNA vaccination in the human skin [52]. Skin is an ideal tissue to trigger immune responses because of the preponderance of immune cells in the subsurface blood vessels [53]. These MNs are composed of a polyelectrolyte multilayer assembly of a charge reversal, pH-responsive copolymer and heparin; DNA polyplexes are also embedded through electrostatic interactions. The charge reversal, pH-responsive copolymer, which is positively-charged at low pH, becomes negatively-charged at physiological pH. This results in the disassembly of MNs and thus releasing DNA vaccine.

**FIGURE 4:**
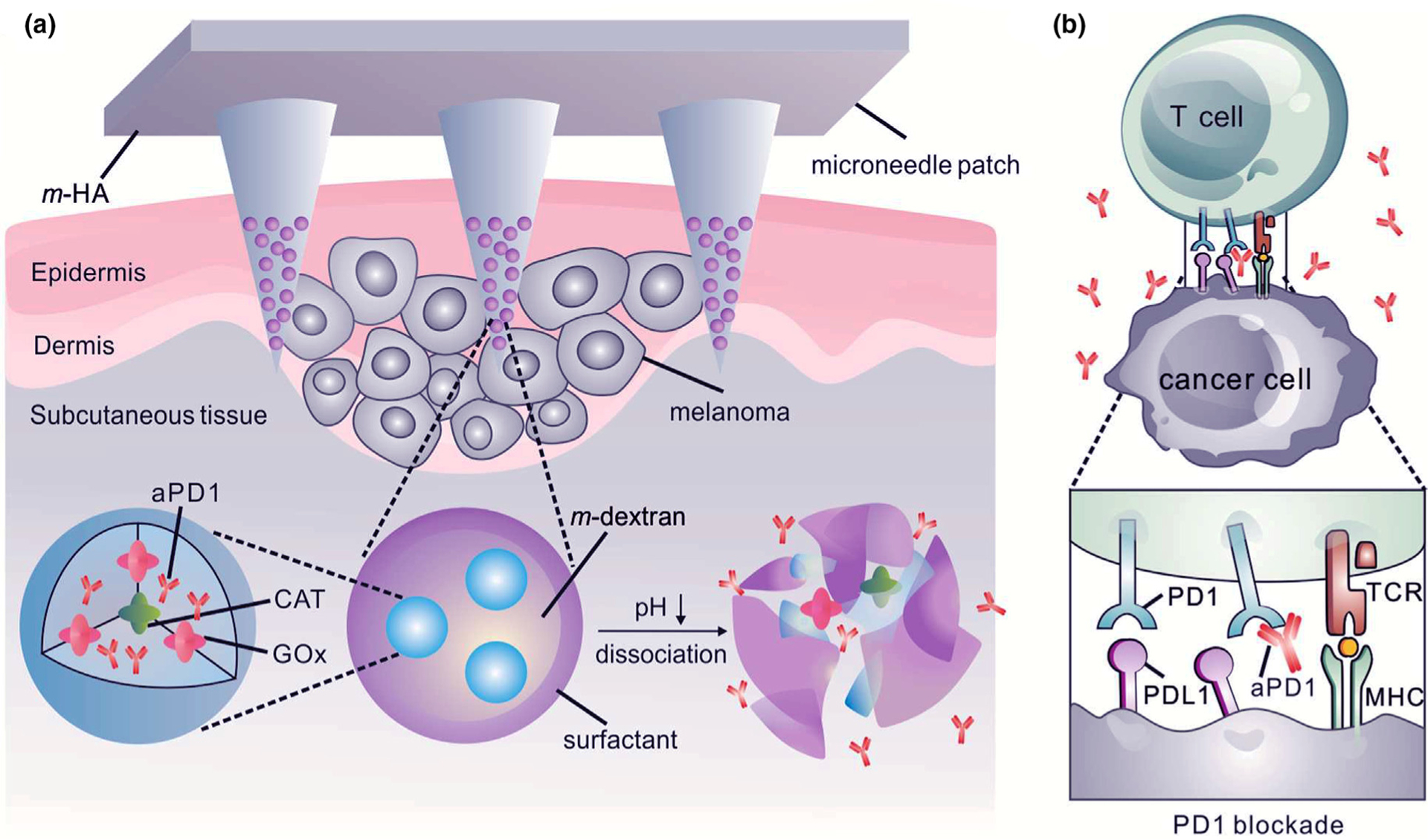
(a) Hyaluronic acid-based microneedles with slow-release of the embedded nanoparticles toward the melanoma site. Nanoparticles are prepared from four components: pH-sensitive polymeric matrix, polyelectrolyte-based surfactant, glucose oxidase/catalyzer (GOx/CAT) enzymatic system, and anti-PD-1. GOx is an enzyme that converts glucose to gluconic acid with the help of the CAT. Accordingly, sensing glucose results in a drop in pH, degradation of nanoparticle and anti-PD-1 release. This gradual release profile ensures potent tumor destructive properties. (b) Schematic depicting the function of anti-PD-1 in preventing T-cell apoptosis by blocking the PD-1 receptor on the T-cell surface. This enables the T-cells to combat tumor cells longer. aPD-1: anti-programmed death-1 protein. Reprinted with permission from [54].

Incorporation of pH-responsive nanoparticles into MNs is another potential route for cancer therapy. For example, hyaluronic acid-based MNs containing pH-sensitive dextran nanoparticles were fabricated to deliver anti-programmed death-1 (aPD-1) antibody [54]. The latter acts as an immune checkpoint inhibitor that prevents T-cells from undergoing apoptosis by blocking the PD-1 receptor on the T-cell surface. This enabled the T-cells to combat tumor cells effectively (Fig. 4). The pH-responsive dextran nanoparticles were prepared using a double emulsion (water-in-oil-in-water) solvent evaporation/extraction approach. The pH-responsive MNs were highly effective in eliminating melanoma cells in a murine melanoma model, compared to systems without triggered release or intertumoral injection.

### Glucose-responsive MNs

When MNs are inserted into the skin, they contact the dermal microcirculation [55] and sense changes in the metabolic contents of the biological environment in real-time [56]. In people with diabetes, blood glucose levels must frequently be monitored so that antidiabetic drugs are provided in a timely manner [57]. To date, convenient and painless insulin administration can be achieved with the use of glucose-responsive MNs [58,59]. Glucose sensing in these devices proceeds *via* the following three approaches [58,59]:

1. Enzyme-catalyzed environmental changes [59]. This method depends on glucose oxidase (GOx) as a catalytic enzyme for converting glucose to gluconic acid [60]. This reaction results in pH decrease, O_2_ consumption (hypoxia), and generation of H_2_O_2_. These changes have frequently been used as the triggers in the glucose-responsive MNs [61].
2. Glucose-binding molecules [58,59]. Glucose-responsive devices have also been prepared using other materials such as lectins. The most common lectin is concanavalin A, a tetravalent binding protein. However, concanavalin A suffers from toxicity and low stability [62,63].
3. Molecular recognition by diol-binding chemical moieties [58,59]. Alternatives to replace natural glucose-detecting compounds (e.g., lectins or enzymes) include synthetic molecules such as phenylboronic acids that can bind to *cis*-1,2 or *cis*-1,3 diols in glucose and other similar diols.

Insulin plays a key role in regulating blood glucose levels in the human body. Physiological release of insulin follows a continuous, complex pattern from *β*-cells within the islets of Langerhans of the pancreas [64]. Insulin is secreted primarily in response to elevated blood glucose concentrations. Basal insulin is secreted 24 h a day, irrespective of whether a person eats. In contrast, bolus insulin is secreted by the pancreas in response to ingestion of food for rapid blood glucose regulation. Insufficient insulin secretion or inadequate reaction between cell receptors and insulin causes glucose metabolic abnormalities that result in diabetes mellitus [65]. People with type I and advanced type II diabetes require frequent insulin injections. However, conventional, open-loop insulin delivery systems fail to precisely control blood glucose levels within a normal range and often result in fatal overdosage. Conversely, closed-loop delivery systems intelligently regulate insulin release to blood glucose concentration changes. These intelligent systems are more desirable for treating diabetes. By integrating MN technology, the closed-loop MN patch systems have become an emerging approach for glucose-responsive insulin delivery [66,67]. Glucose-responsive MNs represent a major share of the bioresponsive MNs.

For the GOx-based MNs, glucose sensing is based on the use of pH, reactive oxygen species (ROS) or hypoxia-responsive materials [57]. In the presence of high glucose concentration, GOx catalyzes the oxidation of glucose, which results in oxygen consumption (hypoxia) as well as the production of gluconic acid (pH decrease) and H_2_O_2_. Each of these variations may be sensed by the related responsive polymer. The sensitive polymer becomes dissociated, dissolved or swollen, enabling the release of insulin [54].

**FIGURE 5:**
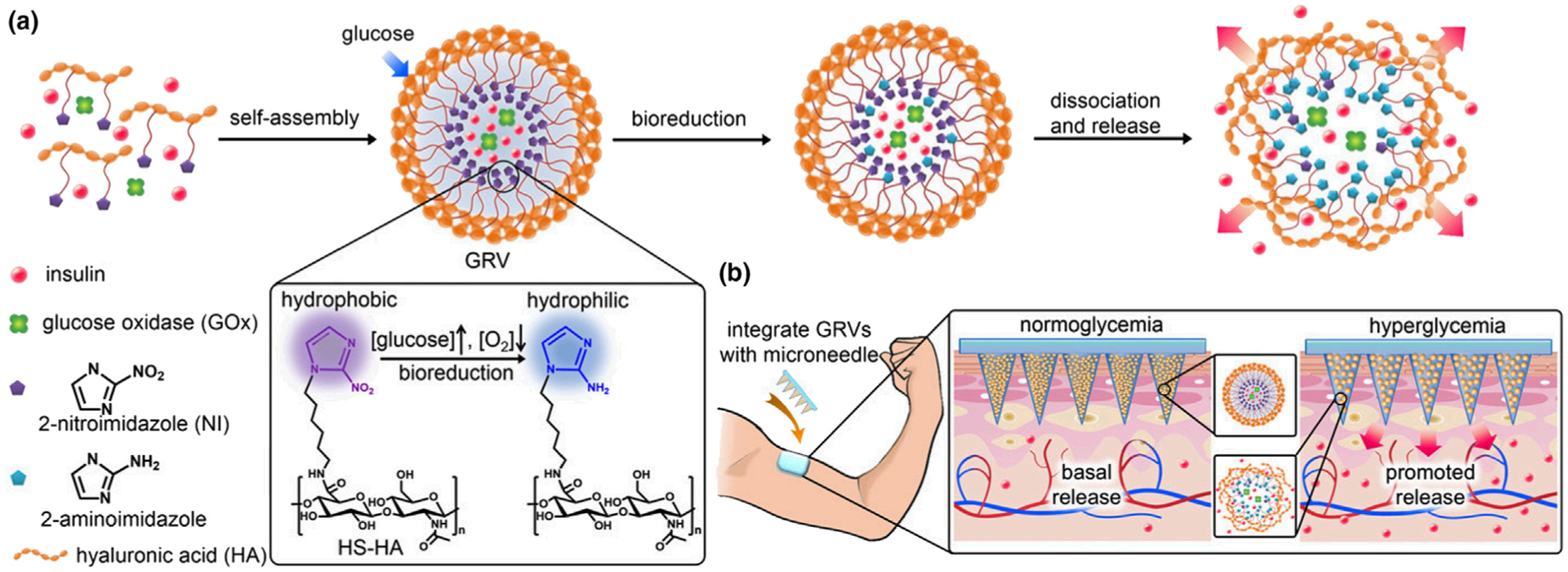
Schematic of the glucose-responsive insulin delivery using hypoxia-sensitive vesicle-loading MN patches. (a) Formation and mechanism of glucose-responsive vesicles (GRVs) composed of hypoxia-sensitive hyaluronic acid (HS-HA). (b) Schematic of the GRV-containing MN-array patch (smart insulin patch) for *in vivo* insulin delivery triggered by a hyperglycemic state to release more insulin. Reprinted with permission from [20].

In the construction of the first prototype of the closed-loop glucose-responsive MNs, Gu and coworkers designed a glucose-responsive MN patch containing glucose-responsive vesicles loaded with insulin and GOx (Fig. 5). The vesicles are formed by hypoxia-sensitive 2-nitroimidazole-modified hyaluronic acid. In the presence of GOx-induced hypoxia that is associated with high blood glucose levels, the hydrophobic 2-nitroimidazole can undergo reduction into hydrophilic 2-aminoimidazole. This transition induces the dissociation of the vesicle and release of insulin. In type I diabetic mice, the MNs were capable of normalizing blood glucose level rapidly and maintained a normal glycemic state for four hours [20].

**FIGURE 6:**
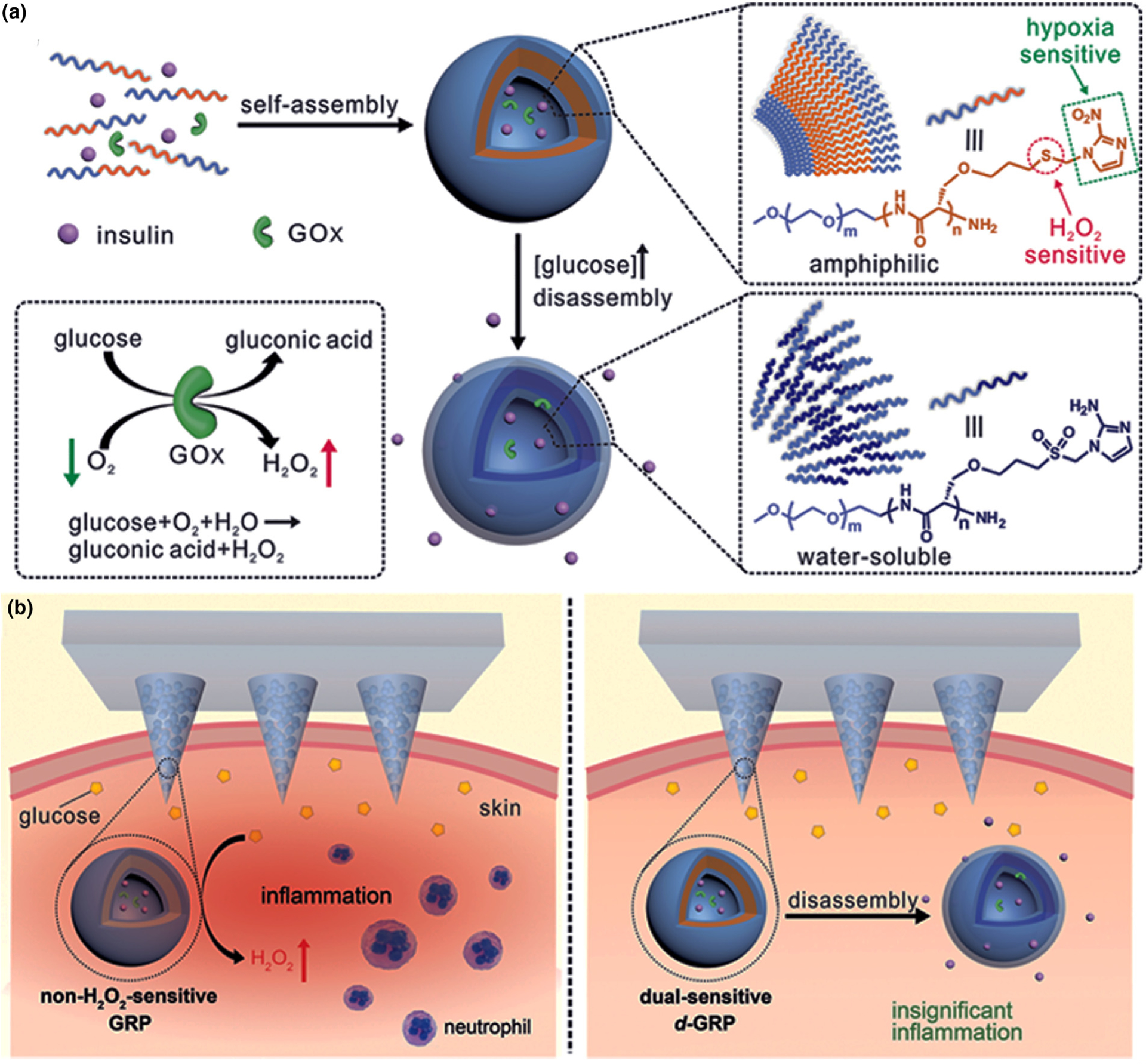
(a) Self-assembled vesicles containing insulin and glucose oxidase (GOx). The dual-responsive system is prepared from poly(ethylene glycol) (PEG) and polyserine diblock copolymer modified with 2-nitroimidazole *via* a thioether [PEG-poly(Ser-S-NI)]. Converting thiol to sulfone is the reason for improved water solubility and higher release of insulin. (b) Comparison between hypoxia-responsive MNs and hypoxia/H_2_O_2_-responsive MNs. The former design causes high free radical levels and thereby cell toxicity. In addition, unwanted inflammation after MN insertion into the skin attracts immune cells. These side effects are addressed using dual-sensitive MNs in the insulin patches. Reprinted with permission from [68].

Polymersome-based vesicles were prepared using a hypoxia/H_2_O_2_-sensitive diblock copolymer to produce a dual-responsive MN patch for glucose-responsive insulin delivery (Fig. 6) [68]. 2-Nitroimidazole was employed as the hypoxia-responsive agent. It was conjugated to the diblock copolymer *via* a thioether moiety. The thioether functions as an H_2_O_2_-sensitive agent that renders the polymer more hydrophilic when it was converted into a sulfone in presence of H_2_O_2_. This amphiphilic polymer was capable of self-assembling into a nanoscale bilayer vesicle (polymersome) for encapsulation of recombinant human insulin and GOx in the aqueous core. When exposed to high blood glucose levels, GOx caused decrease in oxygen concentration and increased in reactive oxygen species levels. When both molecules reached the desired concentration, the vesicles were stimulated to respond by releasing insulin. The H_2_O_2_-sensitive thioether moiety in the vesicle polymer was able to to eliminate H_2_O_2_ to improve the catalytic function of GOx. *In vivo* results showed that blood glucose levels were kept under control for 10 h in type I diabetic mice.

**FIGURE 7:**
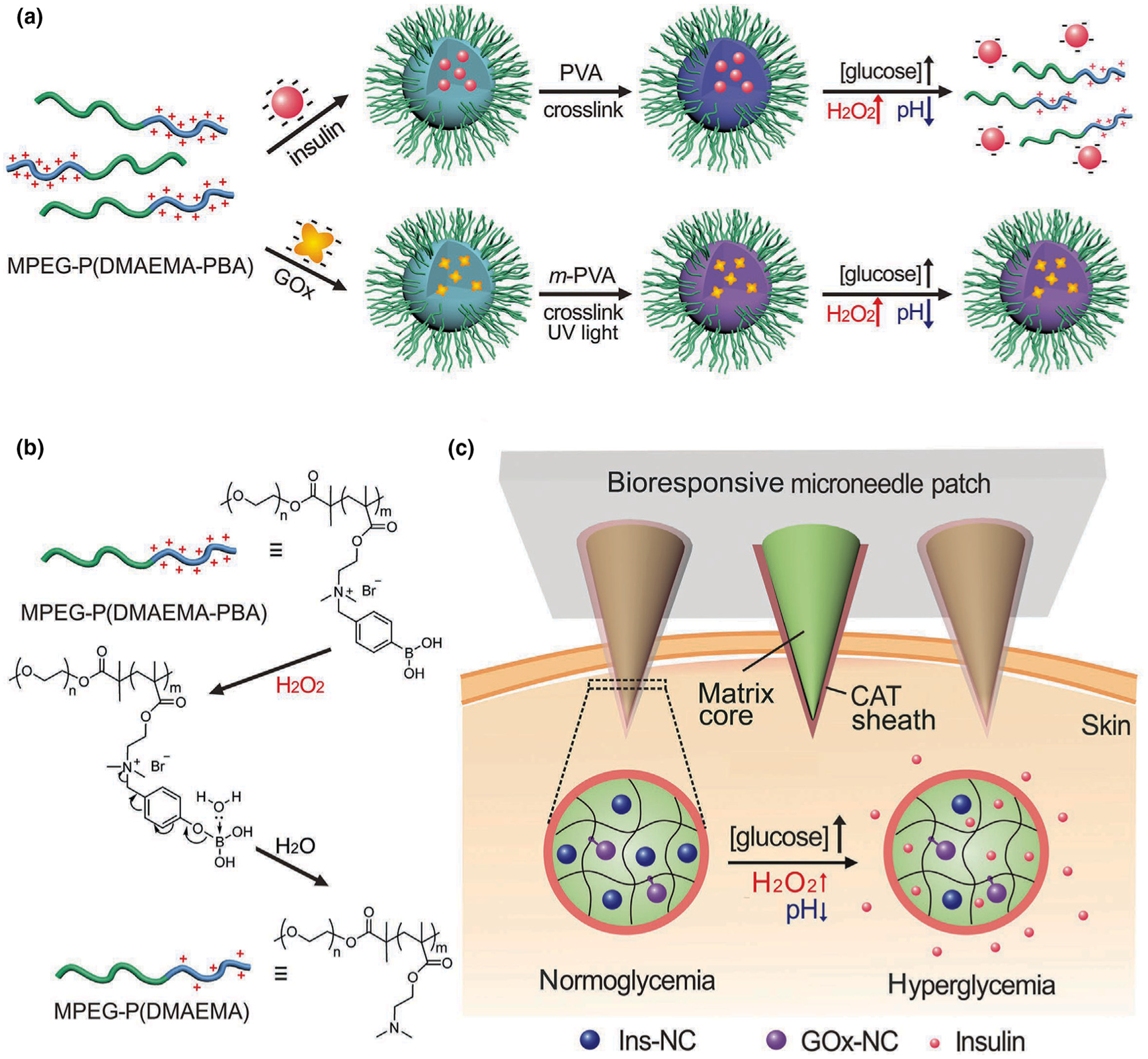
Schematic of a glucose-responsive insulin delivery system utilizing H_2_O_2_ and pH cascade-responsive NC-loading MN patch. (a) Formation of Ins-NCs and GOx-NCs and mechanism of glucose-responsive insulin release. (b) Schematic of H_2_O_2_-triggered charge reduction of the polymer. (c) Schematic of the NC-containing MN patch with a CAT sheath structure for *in vivo* insulin delivery. Insulin release is triggered under a hyperglycemic state. Reprinted with permission from [69].

Nanocomplex micelles (NCs) were used in the design of H_2_O_2_/pH dual-responsive insulin releasing MN-array patches [69]. The difference between this system and others was the encapsulation of insulin and GOx separately in degradable and non-degradable NCs, respectively (Fig. 7). Thus, GOx was preserved after each trigger while a specific amount of insulin is released based on the stimulation level. These NCs were trapped in the core of the microneedle matrix gel, which was covered by a layer of catalase, a H_2_O_2_-scavenging enzyme, which preserved the adjacent tissue from the harmful effect of H_2_O_2_. When the glucose level was elevated, GOx converted glucose into gluconic acid and H_2_O_2_. The elevated H_2_O_2_ converted the disruption of the insulin-NCs and insulin release.

**FIGURE 8:**
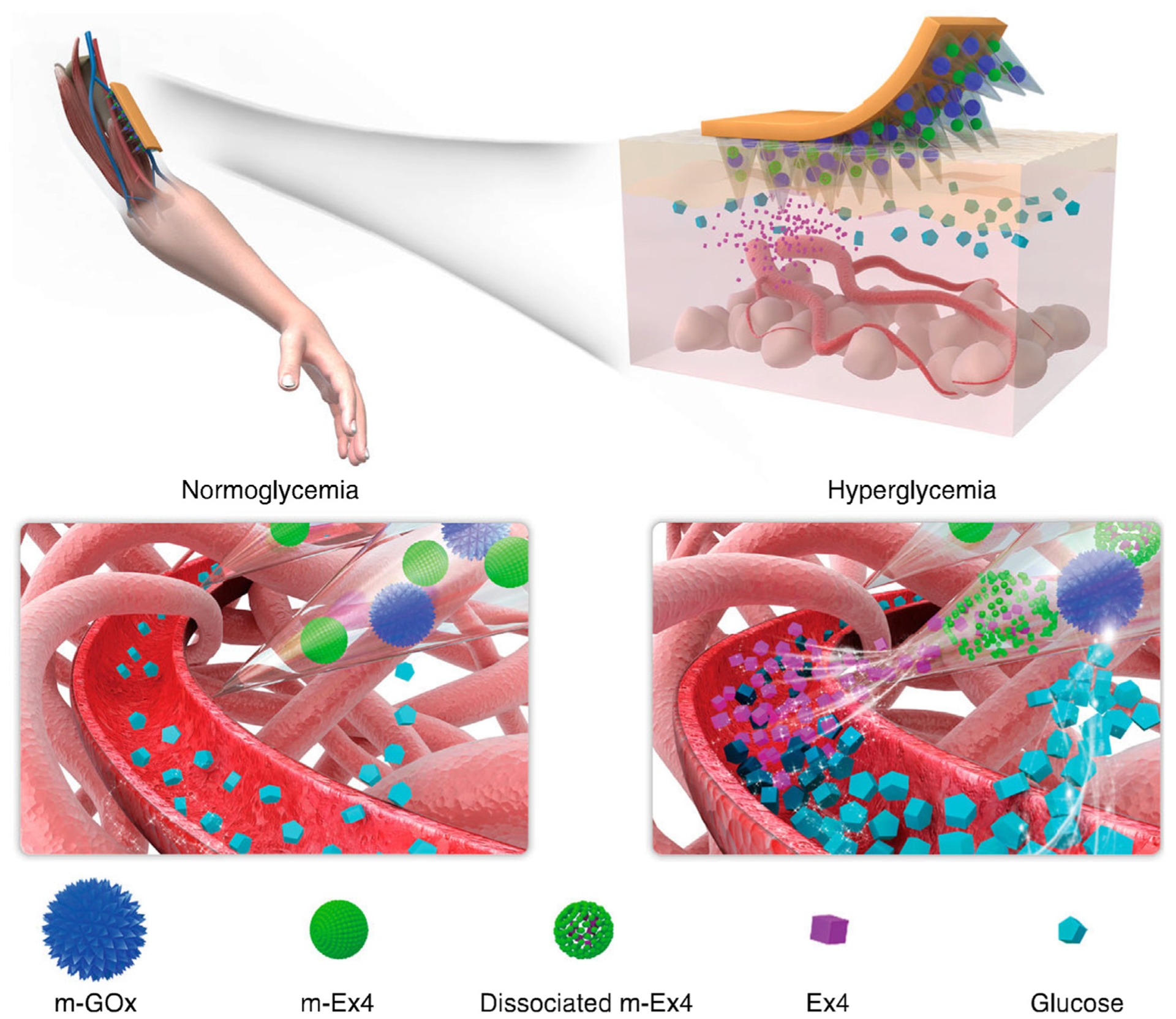
Copper phosphate-mineralized particles containing glucose oxidase (mineralized GOx or m-GOx) and calcium phosphate particles with Ex4 (mineralized Ex4 or m-Ex4) in the MNs. m-GOx is static elements, converting glucose to gluconic acid only. Low pH causes solubilization of pH-sensitive biominerals such as calcium phosphate. This transition causes release of Ex4 and thus reduction in blood glucose levels. Reprinted with permission from [70].

Glucagon-like peptide-1 (GLP-1) receptor agonists have been shown to have a promising effect on type II diabetic patients. Exendin-4 (Ex4), a GLP-1 receptor agonist, is more resistant against degradation and suitable for type II diabetes treatment. A GOx-based glucose responsive MN patch was reported for Ex4 delivery (Fig. 8) [70]. Two different macromolecule-biomineral hybrid particles were used for encapsulation of Ex4 and GOx. In this way, the release of one was not dependent on the other and the Ex4 can be liberated while the GOx particles remain intact. In high blood glucose conditions following by low pH, the Ex4 particle degraded and the drug was released. The combination of MNs with mineralized particles also led to mechanical stability of the MNs, enabling them to be inserted more easily into the epidermis. The resulting patch showed promising long-term release and regulation of blood glucose levels in type II diabetic mice.

Transdermal MNs based on boronic acid, especially PBA, have been used for glucose-sensitive self-regulated insulin delivery. The principle of PBA-mediated glucose-responsive drug delivery is the reversible reaction between PBA and *cis*-diols. Phenylboronic acid is a synthetic Lewis acid that binds reversibly with the hydroxyl groups in glucose to form cyclic esters through its phenyl borate group [57]. This characteristic is utilized in self-regulated delivery based on blood glucose levels. Most PBA moieties are negatively-charged and relatively hydrophilic when the pH is above the pKa of PBA (8.2–8.6). However, once the pH of the solution is below the pKa of PBA, PBA molecules become neutral and hydrophobic. Changes in hydrophilicity of PBA-functionalized materials induce swelling or shrinking of PBA-based platforms, which, in turn, affects drug release profile [57,72]. A limitation in PBA-based insulin delivery is that glucose-responsiveness is only apparent at pH values above the pKa of PBA. To overcome this limitation, PBA has been often modified to reduce the pK_a_ and enable PBA to function around physiological pH [73,74]. In the presence of glucose, negatively-charged PBA is dominant [75]. As a result of repulsion between charges, the polymers swell or disintegrate to release insulin [76].

**FIGURE 9:**
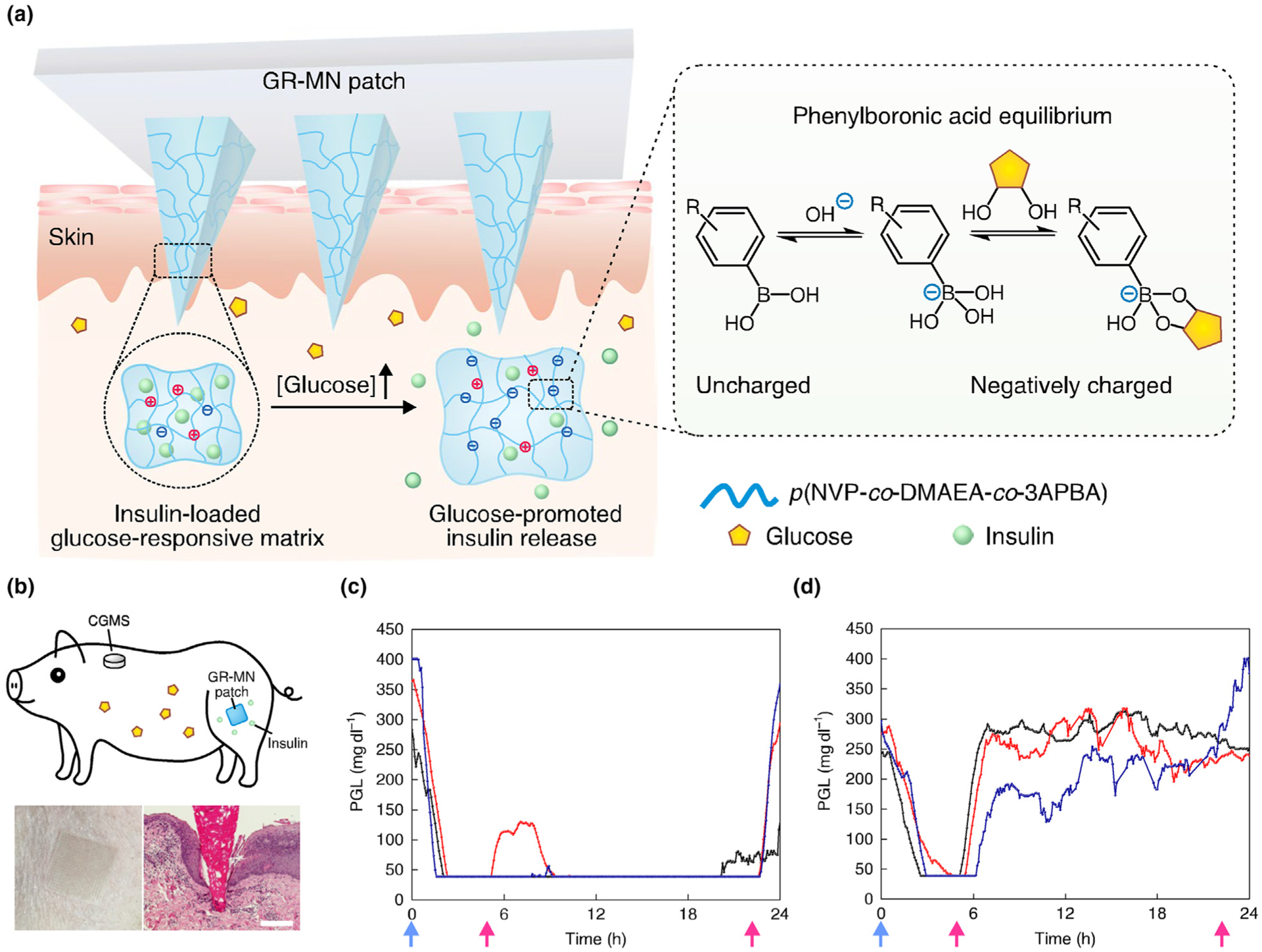
(a) Polymeric MN composed of a polymeric matrix derived from poly(*N*-vinylpyrrolidone-*co*-2-(dimethylamino) ethyl acrylate-*co*-3-(acrylamido)PBA), insulin and a crosslinker (ethylene glycol dimethacrylate (EGDMA)). Insulin and phenylboronic acid (PBA) are dispersed in the polymeric matrix, with higher capacity for insulin storage. The entire MN volume acts as a closed-loop delivery system. Inset shows the mechanism in which glucose binds PBA via a reversible reaction. (b) Top: schematic of a minipig treated with a glucose-responsive microneedle (GR-MN) patch at the leg site and monitored with a continuous glucose monitoring system (CGMS). Bottom left: photograph of a GR-MN patch applied on a minipig’s leg. Bottom right: haematoxylin and eosin-stained section of minipig skin penetrated by one microneedle. Scale bar: 200 μm. (C, D) Plasma glucose levels (PGLs) in streptozotocin-induced diabetic minipigs (*n* = 3) after treatment with GR-MN (c) and non-responsive crosslinked microneedle (CR-MN) patches (d). Reprinted with permission from [71].

A PBA-based glucose-responsive MN system has recently been reported for experimental treatment of diabetes. In the presence of a blood glucose level, glucose can bind to PBA molecules, inducing increased negative charge within the MN matrix. This negative charge stimulates insulin release via two mechanisms (Fig. 9) [71]. In the first mechanism, electrostatic repulsion causes a volume increase in the MNs, which, in turn, induces increased diffusion of insulin. In the second mechanism, the electrostatic attraction between insulin and the matrix is decreased, which leads to increased insulin release. The glucose-responsive MNs were capable of maintaining blood glucose in a normal range for nearly one day in minipigs.

Hypoglycemia represents an inherent risk in open-loop insulin delivery [78]. Glucose-responsive MNs have been fabricated to release native glucagon. This helps to prevent the development of hypoglycemia that results from excessive insulin administration beyond the required dosage [72]. Native glucagon was dispersed in an MN matrix comprising photo-crosslinked methacrylated hyaluronic acid. Glucagon is sensitive to chemical degradation and physical denaturation in physiological conditions. Hence, a stabilizing cover is required in addition to encapsulation. During hyperglycemia or euglycemia, the 4-acrylamido-3-fluorophenylboronic acid (AFBA) component of the formed, which resulted in microgel swelling. During hypoglycemia, the concentration of glucose fell below that of AFBA. Biscomplexation of AFBA with glucose creates secondary crosslinking between the polymer segments. This interaction resulted in shrinkage of the microgel and release of the loaded glucagon. The MNs demonstrated effective prevention of hypoglycemia when applied transdermally on diabetic rat skin.

**FIGURE 10:**
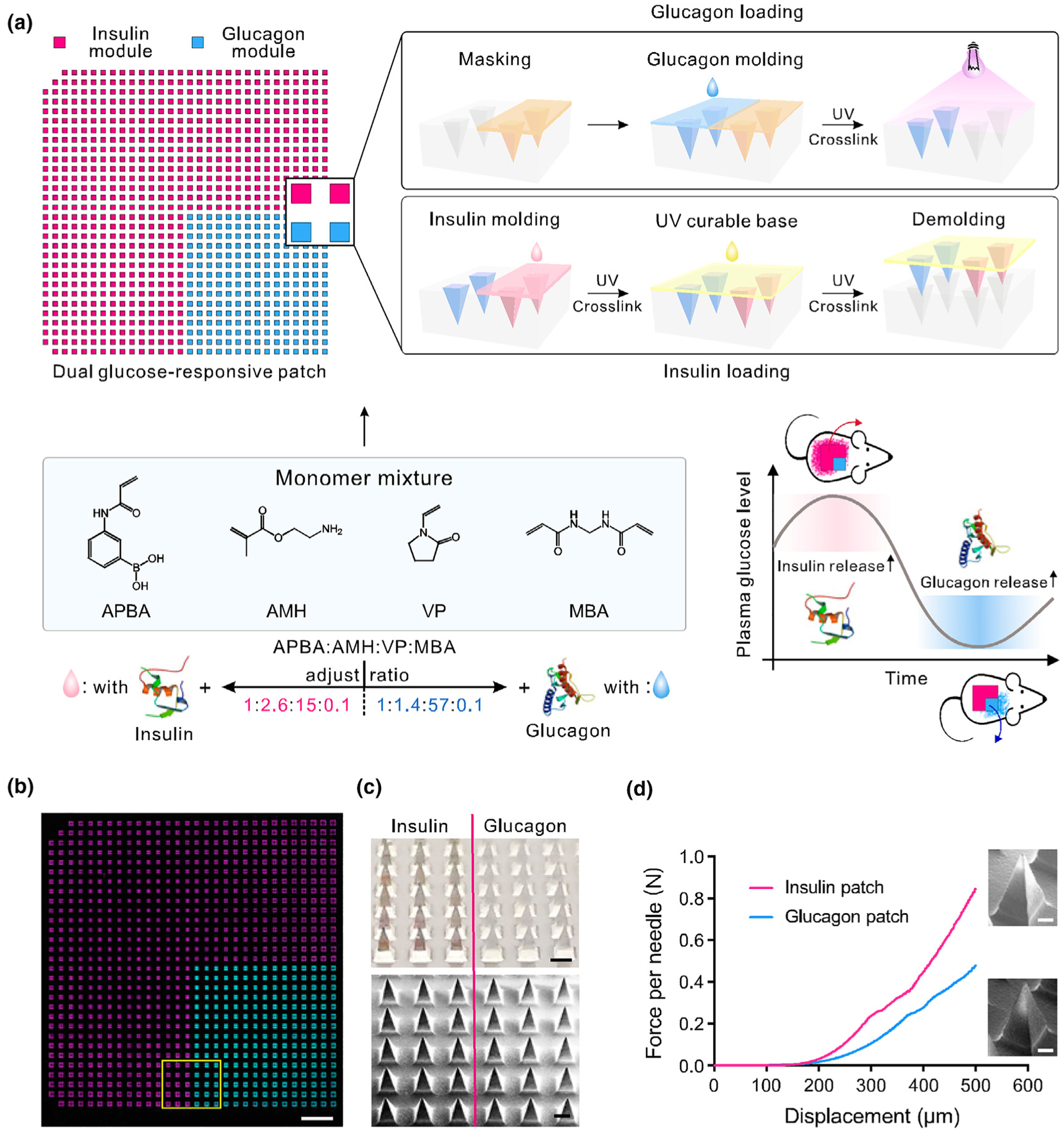
Schematic and characterizations of the dual glucose-responsive hybrid microneedle-array patch. (a) Schematic illustration of the fabrication process and glucose-responsive glycemic control mechanism of the hybrid patch. Insulin or glucagon release can be promoted by hyperglycemic and hypoglycemic conditions, respectively. The missing microneedles at two corners of the mold are used for orientation tagging. (b) A tile-scanned fluorescence microscopy top-view image of the FITC-labeled glucagon (cyan) and Cy5-labeled insulin (magenta) hybrid microneedle patch. (Scale bar, 2 mm.) (c) Photograph (Top) and SEM image (Bottom) of the MN patch at the intersection. (Scale bar, 300 μm.) (d) Mechanical performance of the glucagon and insulin MNs, respectively. A representative enlarged MN SEM image is placed next to each curve. (Scale bar, 100 μm.) Reprinted from [77] with permission from PNAS.

More recently, Gu and coworkers reported a hybrid glucose-responsive patch as a synthetic artificial pancreas to deliver insulin and glucagon in a glucose-dependent manner (Fig. 10) [77]. This patch was composed of dual modules that contain either insulin or glucagon, thereby mimicking the function of pancreatic islet cells for comprehensive regulation of blood glucose levels. The modules were photo-polymerized from the same monomers (1-vinyl-2-pyrrolidinone (VP), 2-aminoethyl methacrylate hydrochloride (AMH), and 3-(acrylamido) phenylboronic acid (APBA)) but with different monomer ratios, so that the resulting modules can be “dually responsive” to either hyper- or hypoglycemic conditions to achieve differently payload release profile. The dual glucose-responsiveness of insulin and glucagon delivery can be ascribed to the synergistic net charge changes of the AMH/APBA polymeric matrix at various glucose concentrations, the shrinkage or swelling of the matrix, and the difference in isoelectric points of the insulin and glucagon molecules at physiological pH. In type I diabetic mice, the hybrid patch can effectively control hyperglycemia and mini mize the risk of hypoglycemia in the settings of insulin therapy with insulin overdose or simulated delayed meal.

**FIGURE 11:**
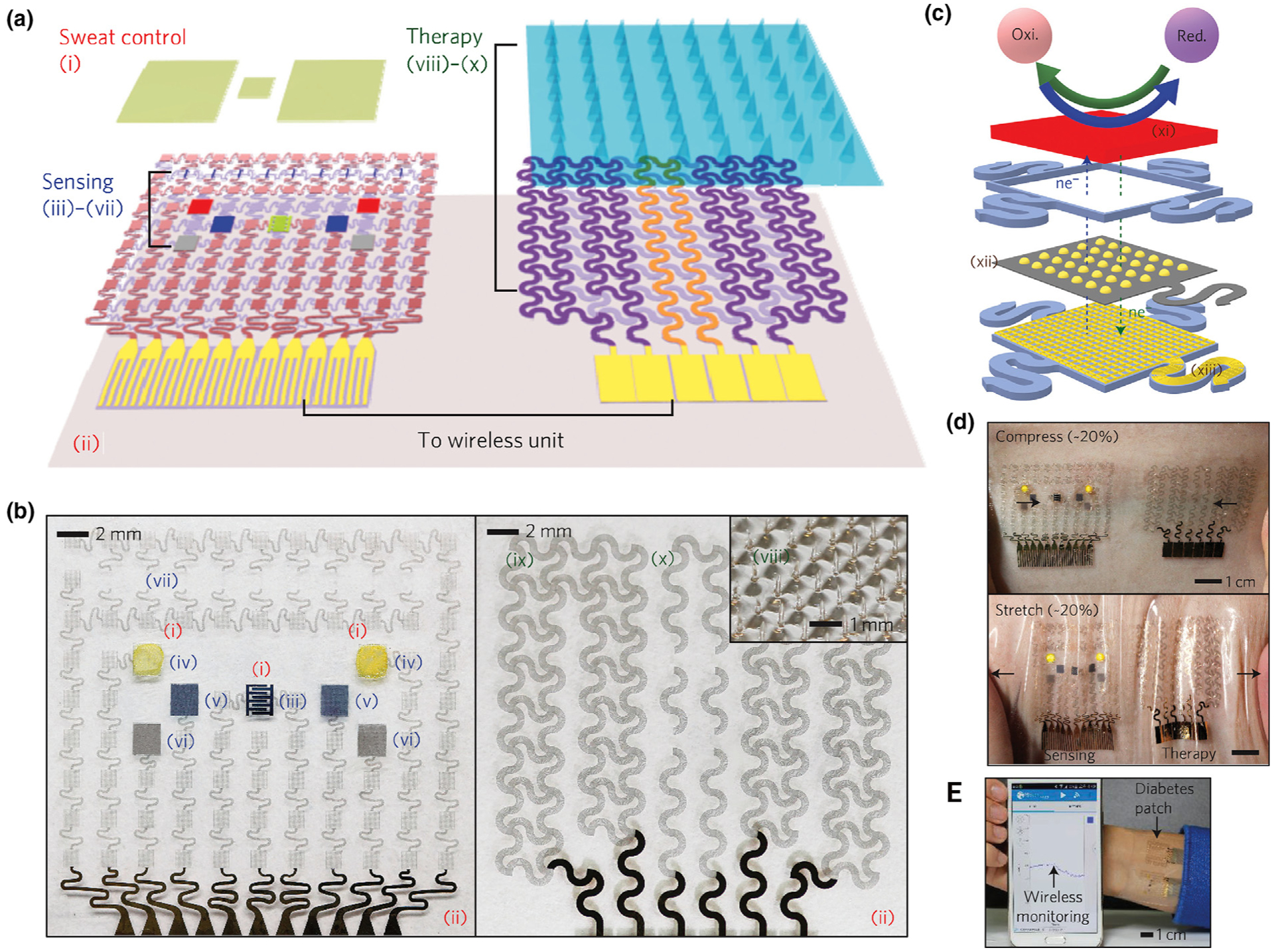
Schematic and corresponding images of the graphene-hybrid electrochemical devices and thermoresponsive drug delivery microneedles. (a), Schematic of the diabetes patch, which is composed of the sweat-control (i, ii), sensing (iii–vii) and therapy (viii–x) components. (b) Optical images of the electrochemical sensor array (*left*), therapeutic array (*right*) and magnified view of the drug-loaded microneedles (inset). (i) sweat-uptake layer (Nafion); (ii) water-proof film (silicone); (iii) humidity sensor (poly(3,4-ethylenedioxythiophene); PEDOT); (iv) glucose sensor (Prussian blue; PB); (v) pH sensor (polyaniline; PANi); (vi) counter electrode (Ag/AgCl); (vii) tremor sensor (graphene); (viii) microneedles with drugs (polyvinyl pyrrolidone@ tridecanoic acid); (ix) heater (Au mesh/graphene); (x) temperature sensor (graphene). (c) Schematic of the graphene-hybrid electrochemical unit, which consists of electrochemically active and soft functional materials (xi), gold-doped graphene (xii) and a serpentine Au mesh (xiii), from top to bottom. (d) Optical images of the diabetes patch laminated on human skin under mechanical deformations and wireless monitoring *via* Bluetooth connection. Reprinted with permission from [80].

A closed-loop diabetic patch system capable of simultaneously sensing and delivery has been developed to adjust the body’s glucose level. The device can measure the level of glucose in human blood to monitor the delivery of a specific amount of insulin [79]. It is envisaged that polymeric MN patches that are wirelessly connected to a unique smart phone will be available soon for personalized patient treatment. These MNs automatically regulate a process variable (e.g., glucose or cholesterol level) to the desired state. A flexible device based on functionalized chemical vapor deposition of graphene has been developed for simultaneous monitoring of diabetes and delivery of therapeutics [80]. The device comprises (i) sensors for monitoring temperature, humidity, glucose concentration and pH, (ii) a heater, and (iii) polymer-based MNs that can be thermally activated to release drugs/biomolecules transcutaneously. The continuous monitoring of biomarkers and physiological cues on transcutaneous drug/biomolecule delivery potentially provides a closed-loop, point-of-care treatment for diabetic patients for personalized medicine. Practical applicability is further improved by Bluetooth wireless connection that can report data for analysis (Fig. 11).

### Reactive oxygen species-responsive MNs

Reactive oxygen species (ROS) are the reduction products of oxygen [81,82]. Internal ROS are generated in the mitochondria and play important physiological roles in cellular signaling and metabolism [83]. Abnormal ROS levels are associated with many diseases, which motivates researchers to design ROS-responsive MN systems for management of those disease. For instance, ROS-responsive MNs have been prepared for reducing the side effects of *Acne vulgaris* by cross-linking poly(vinyl alcohol) using a dual phenylboronic acid-contained linker [84]. Reactive oxygen species-responsive materials may be prepared by incorporating ROS moieties such as thioether [85], selenium [86], tellurium [87], thioketal [88], oxalate [89] and phenylboronic acid/ester [90] into the polymer chains. These materials are synthesized through step-grow and/or ring-opening polymerization of ROS moiety-containing monomers, or through post-polymerization modification to integrate ROS moiety-containing molecules [91].

Reactive oxygen species are highly concentrated in pathological conditions such as cancer, stroke, arteriosclerosis and tissue injury because of disruption in oxidation–reduction balance. Hence, ROS can be used as markers to identify the site of action for targeting treatment [13,91]. The ROS-responsive polymers can be divided into two groups: ROS-induced non-cleavable hydrophobic–hydrophilic transition and ROS-induced structural cleavage. In the first category, ROS oxidize chalcogen elements (e.g., S, Se and Te) to alter their valence. Oxygen can bind with chalcogen elements while polarized groups provide hydrogen to bind with water. As a result, the material undergoes a change in its hydrophilicity/hydrophobicity. In the second category, ROS react with polymers and result in chemical bond cleavage. Common ROS-responsive polymers include thioketals, phenylboronic acids/esters (PBAs/PBEs), vinyl di-thioethers and proline oligomers [91]. Among these compounds, PBAs/PBEs are the most used ROS-responsive polymers in MN systems.

**FIGURE 12:**
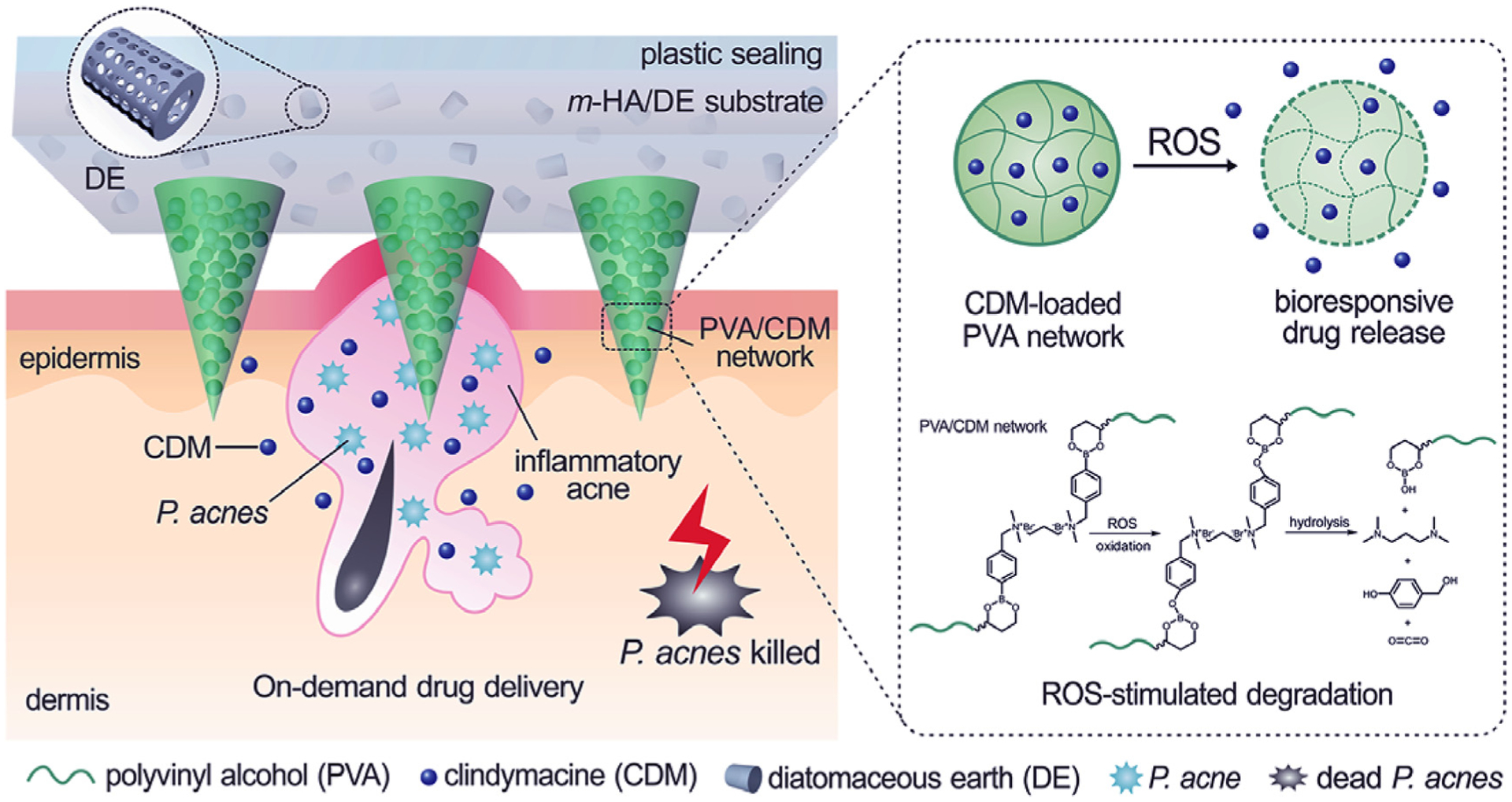
ROS-responsive MNs that release clindamycin inside the skin when they come into contact with high concentrations of ROS, which may be as high as 500 μM in acne inflammation. The MNs are prepared from poly(vinyl alcohol) that is crossed-linked with a phenylboronic acid-containing linker. Oxidation and hydrolysis reaction by ROS degrades the polymer matrix and releases the antibiotic. Reprinted with permission from [84].

Because the level of ROS augments significantly in inflammation, ROS-responsive MNs are useful for combating inflammation. For example, the antibiotic clindamycin was loaded into ROS-responsive polymeric MNs. The ROS-responsive MNs were capable of penetrating the outer layer of the skin, releasing the target drug at the specific site of inflammation in the designated therapeutic range [84]. ROS-responsive MNs were used for topical treatment of inflammation caused by *Propionibacterium acnes*, a Gram-positive human skin commensal that is involved in the pathogenesis of acne [92]. Fig. 12 illustrates ROS-responsive MNs, with targeted antibiotics release, that are capable of overcoming the obstacles for acne treatment. Despite improvements in the dermal and oral routes of administration of anti-acne medications, there are obstacles in treating these skin lesions. These limitations arise from the low penetration of antibiotics through the *stratum corneum*, as well as the potentially harmful effects associated with the use of high doses of antibiotics [93,94].

### Enzyme-responsive MNs

Enzymes are involved in almost all metabolic reactions in the body and have vital roles in the maintenance of a healthy body. Many diseases are related to abnormality in the function of enzymes or alteration in the amount of enzymes [13]. Tumors, in particular, have abnormal enzyme expression profiles in each stage of cancer, which may be used as markers for identification and therapy [47,95].

The hyaluronic acid family of molecules, in particular hyaluronidase, has recently been shown to be potential prognostic biomarkers and therapeutic targets in the treatment of cancer [96]. For instance, it has been shown that the combination of aPD1 and 1-MT has a synergistic anti-tumor effect. The presence of hyaluronidase in the tumor microenvironment elevates microneedle degradation and provides the targeted anti-tumor effect in mice. In the presence of hyaluronidase, release of aPD1 and 1-MT increased to 4 and 2 times, respectively, compared with the absence of hyaluronidase [97].

Heparin, a common anticoagulant, is routinely administered to prevent coagulation in thrombosis. However, systemic or local delivery of anticoagulants remains challenging for precise anticoagulant regulation. Under-dosage or over-dosage could lead to dangerous consequences caused by rapid clearance from the body or spontaneous bleeding complications. To address this issue, a feedback-controlled anticoagulant system based on thrombin-responsive polymer-drug conjugates has been developed [98]. Thrombin plays an important role in blood coagulation by producing insoluble fibrin from fibrinogen [99]. A thrombin-cleavable peptide was introduced as a linker between heparin and hyaluronic acid. The peptide was cleaved when thrombin is activated, which triggered the release of heparin from the thrombin-responsive heparin-conjugated hyaluronic acid (TR-HAHP) matrix. When the thrombin concentrations elevated, heparin was rapidly released from the TR-HAHP matrix. The released heparin was able to inhibit coagulation activation by inactivating thrombin [98].

### Thermosensitive MNs

Thermosensitive polymers undergo phase transition (sol–gel or vice versa) based on changes in human body temperature [100,101]. Different types of polymers have been used in thermosensitive MN devices and most of them undergo gel-sol transition to be solubilized for the release of the incorporated drugs. For example, chitosan and *β*-sodium glycerophosphate (*β*-GP) were incorporated in MN formulations to enhance the dissolution capability of the MNs [102]. The gel made of chitosan and *β*-GP is reversible; when the temperature is higher than the phase transition temperature of chitosan and *β*-GP, the hydrophobicity of chitosan molecules increases and the hydrogen bonding weakens, resulting in gel formation. This characteristic may be used to accelerate the rate of dissolution of MNs prepared from dextran. Other thermosensitive MNs may incorporate amphiphilic block copolymers such as polyethylene oxide-polypropylene oxide copolymers (poloxamers or pluronics, PPO-PEO-PPO). These amphiphilic block copolymers are capable of undergoing thermal sol–gel transition [103–105].

Thermosensitive polymers such as Pluronic undergo phase transition from sol-gel or vice versa when triggered by human body temperature [100,101,106]. They can be applied as a solution into the skin and transform into a gel. Microneedles may be used to create holes in the skin for deposition of the thermosensitive polymer solution. Upon deposition into the skin, the polymer solution converts into a gel (sol–gel transition). For example, maltose MNs were employed to create micropores in the skin and methotrexate-loaded solution was used to fill the pores. The solution transformed into a gel at skin temperature and released the cargo in a sustained manner [107]. Hydrogels based on thermosensitive poly(lactic-co-glycolic acid)-polyethylene glycol-poly(lactic-co-glycolic acid) copolymers have been utilized with MNs for the delivery of plasmid deoxyribonucleic acid into the skin [108]. In addition, thermosensitive polymers may be utilized to increase the dissolution capability of MN polymer matrices (gel-sol).

## Future perspectives and clinical translation

Stimuli-responsive microneedle patches represent an emerging technology for on-demand drug delivery to improve treatment efficacy and alleviate systemic side effects. As described above, such systems can be triggered by a variety of physiological or external stimuli. Microneedles triggered by physiological signals are also capable of self-regulated/closed-loop drug delivery in response to those physiological signals, thereby maximizing therapeutic efficacy and potentially enhancing patient compliance [109]. Although MNs triggered by external physical stimuli are highly robust, they often require an external source. Portable units for the administration of these external stimuli may not be readily accessible, and could be excessively costly for patients.

**FIGURE 13:**
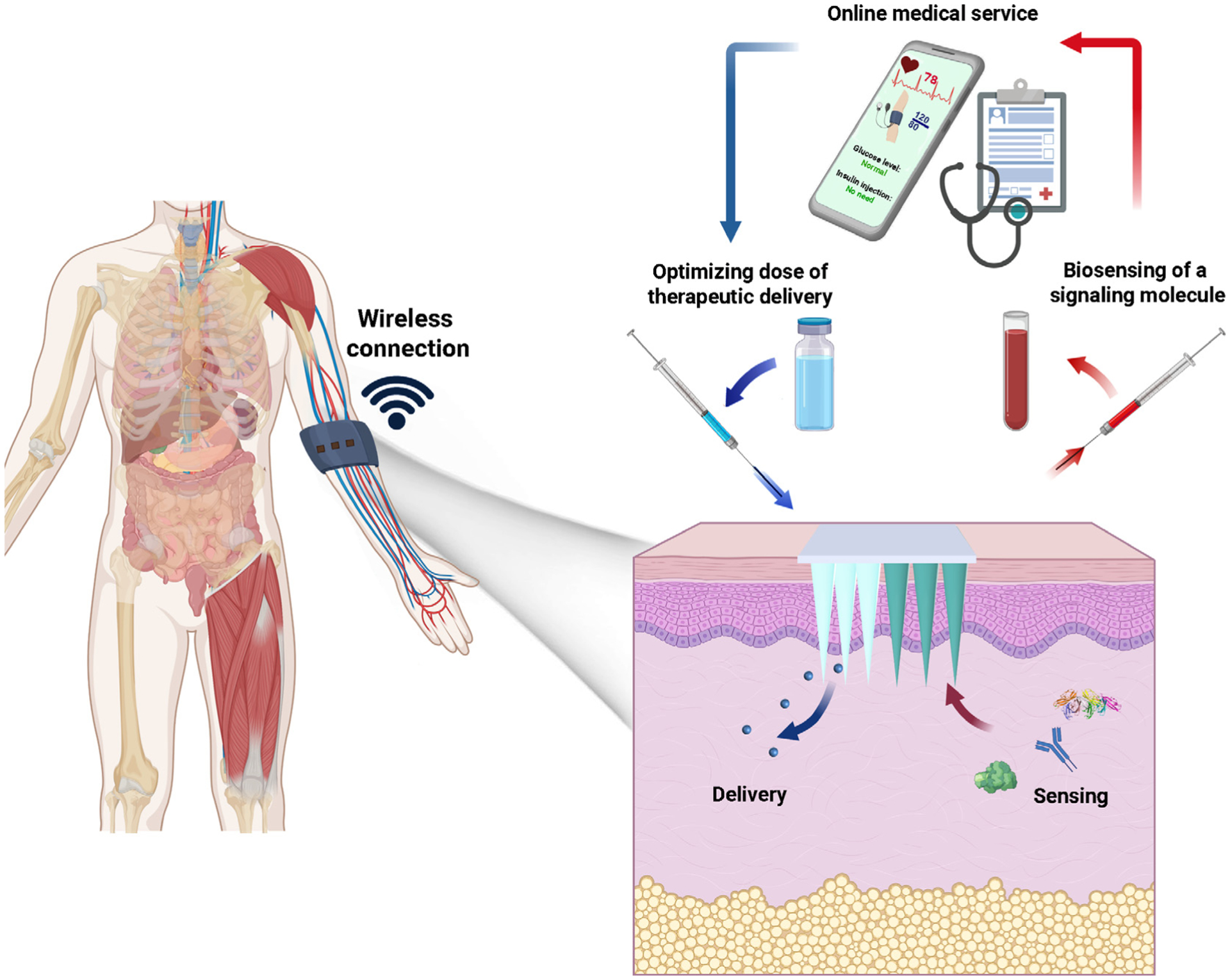
Bioresponsive MNs consist of two major components. The first component comprises a sensing system which is responsible for monitoring environmental signals. The second component comprises a delivery system which releases therapeutic agents based on signals collected from the sensing system. The MN patch may be wirelessly connected to a smartphone for reporting and data analysis.

Bioresponsive systems, on the other hand, generally comprises a monitoring component to sense the surrounding conditions, and an actuator part with the capability to trigger drug release (Fig. 13). Because of the presence of biosensing functions, it is possible to control payload release at or above a certain biosignal concentration/threshold, as well as inhibit the release when the biosignal level is in its normal range [110,111]. These MNs enable point-of-care continuous monitoring and delivery. However, further thorough validation in different animal or prelincal models is still needed. These delivery systems potentially incorporate an embedded or external control system that is connected to smart devices to report the progress to the patients’ physician for personalized medical applications [112].

From a regulatory point of view, most of the current applications need to undergo rigorous clinical trials. Although only a limited number of MN systems will ultimately reach the market, the different phases of clinical trials ensure the safety and effectiveness of stimuli-responsive materials. In addition, since most of the reported applications fall in the pharmaceutical field, they require good manufacturing practice audits. Injector-based devices are recognized as second class medical devices, which include hollow MN-based insulin delivery systems [78,92,113]. Meanwhile, clinical trials have been conducted on dissolving MNs for vaccine delivery [114] as well as MNs for the delivery of various drugs and proteins [115]. Clinical trials on more recently-introduced stimuli-responsive MNs are anticipated to initiate in the near future. Microneedles are expected to offer desired alternative approach for many clinical practices. From a technological point of view, the microelectronic field has the capability of manufacturing very complex micro-devices. These very complex products, which require very expensive production lines, can only be justified when there is a heavy demand to dampen production cost. Another important aspect to be taken into account is the loading capacity. Considering the limited volume available within MN patches when compared to the more widely used hypodermic syringe, an MN design should take into account animal-human translation factors in the dosages. In case the tool cannot guarantee such a translation, an alternative solution is the integration of an ad hoc reservoir with the MN device, as already realized in some cases.

## Figures and Tables

**TABLE 1:** Representative stimuli-responsive microneedle patches regulated by external triggers and their biomedical applications.

Microneedle	Payload	Stimulus	Application	Ref.
Alginate hydrogel matrix coated with poly(lactide)	Peptide-nucleic acid	UV-Light	miRNA sampling and detection	[116]
Poly (2-Hydroxyethyl methacrylate-co-ethylene glycol dimethacrylate)	Ibuprofen	UV-Light	Pain relief	[16]
Hyaluronic acid/PEGlyated gold nanorod	Doxorubicin	Near-Infrared	Cancer therapy	[117]
Polycaprolactone/LaB_6_@SiO_2_	Doxorubicin	Near-Infrared	Cancer therapy	[17]
Poly(l-lactide) matrix coated with PEGylated gold nanorod and micelles	Docetaxel	Near-Infrared	Cancer therapy	[118]
Hyaluronic acid with gold nanocage	Doxorubicin	Near-Infrared	Cancer therapy	[28]
Polyvinylpyrrolidone with polydopamine/lauric acid-coated SiO_2_	Metformin	Near-Infrared	Type II diabetes	[23]
Chitosan/graphene assembled in porous carbon nanocomposites	Cephalexin	Electric	Antimicrobial therapy	[119]
PEG-PEO-PEG triblock copolymer	Methotrexate	Thermal	Cancer therapy	[107]
Polyvinylpyrrolidone with graphene doped with gold and combined with a gold mesh	Metformin	Electrical/thermal	Diabetes	[80]
Polycaprolactone as tip and polyvinyl alcohol/polyvinylpyrrolidone as base	Metformin	Mechanical/thermal	Diabetes	[25]
Poly(caprolactone) as tip and polyvinyl alcohol/polyvinylpyrrolidone as base	Metformin	Mechanical/near-infrared	Diabetes	[40]
Hyaluronic acid as tip and polycaprolactone as base	Vaccine antigens derived from canine influenza virus	Mechanical	Influenza vaccination	[120]

**TABLE 2:** Summary of representative bioresponsive microneedles and their biomedical applications.

Microneedle	Payload	Stimulus	Application	Ref.
Hyaluronic acid with hypoxia-responsive vehicles	Insulin	Glucose/hypoxia	Diabetes	[20]
Photopolymerized *N*-vinylpyrrolidone (NVP), dimethylamino-ethyl acrylate (DMAEA), 3-acrylamido-phenylboronic acid and ethylene glycol dimethacrylate	Insulin	Glucose	Diabetes	[71]
Polyvinylpyrrolidone loaded with mesoporous silica nanoparticles	Insulin	Glucose/ROS	Diabetes	[121]
Hyaluronic acid with H_2_O_2_-responsive vesicles	Insulin	Glucose/ROS	Diabetes	[122]
Hyaluronic acid with pH/H_2_O_2_-dual responsive polymersomes	Insulin	Glucose/ROS/hypoxia	Diabetes	[68]
Polyvinylpyrrolidone with charge-switchable particles	Insulin	Glucose	Diabetes	[123]
ROS responsive poly(vinyl alcohol) hydrogel	Insulin	Glucose/ROS	Diabetes	[63]
Hydrogel containing PBA domain and silk fibroin	Insulin	Glucose	Diabetes	[55]
Hyaluronic acid	Glucagon	Glucose	Insulin-induced hypoglycemia	[72]
Alginate-based MNs with mineralized particles	Exendin-4	Glucose	Type II diabetes	[70]
Hyaluronic acid with dextran nanoparticles	Rosiglitazone	Glucose	Obesity	[124]
Poly(vinyl alcohol) (PVA) with calcium phosphate nanoparticles	3,3′,5,5′tetramethylbenzidine and HRP	Glucose/pH	Glucose sensing	[125]
Hyaluronic acid containing dextran nanoparticles	aPDL1 and IDO	Glucose	Cancer immunotherapy	[97]
Dimethylmaleic anhydride-modified polylysine	p53-DNA	pH	Cancer therapy	[50]
Polycarbonate conjugated poly(*β*-amino ester urethane)	Adjuvants and DNA vaccines	pH	Cancer immunotherapy	[51]
Heparin conjugated hyaluronic acid	Heparin	Thrombin	Blood coagulation	[98]
Polyvinyl alcohol as tip and hyaluronic acid/diatomaceous earth as base	Clindamycin	ROS	Acne vulgaris treatment	[84]
Poly(vinyl alcohol)/Polyvinylpyrrolidone hydrogels embedded with poly (caprolactone) nanoparticles	Carvacrol	Bacterial lipases	Wound repair	[126]
